# Coping strategies mediate the relationship between fear of cancer recurrence and quality of life in postoperative patients with prostate cancer: a multicentre survey

**DOI:** 10.1186/s12894-024-01428-5

**Published:** 2024-03-01

**Authors:** Chen Yu, Zhu Jingzhen, Zhou Luqiang, Yuan Xiaojuan, Zheng Ji

**Affiliations:** Department of Urology, Xinqiao Hospital, The Army Medical University, Chongqing, 400037 China

**Keywords:** Coping, Fear of cancer recurrence, Prostate cancer, Quality of life

## Abstract

**Purpose:**

The aim of the present study was to investigate the relationships between fear of cancer recurrence and quality of life in patients with prostate cancer. A model based on Lazarus’ and Folkman’s theory tested the specific hypothesis: fear of cancer recurrence has a direct and indirect effect on quality of life mediated by coping strategies.

**Methods:**

A questionnaire survey was conducted on 305 patients with prostate cancer who underwent radical surgery, including demographic information, FoP-Q-SF (Fear of Progression Questionnaire), MCMQ (The Medical Coping Modes Questionnaire), QLQ-C30 (Questionnaire for Quality of Life Assessment in patients with cancer, version 3.0), and a mediator model was tested using the PROCESS macro for SPSS.

**Results:**

The total FoP-Q-SF score of 305 postoperative prostate cancer patients was 34.3 ± 5.856, with approximately 41.6% of the patients scoring higher than 34. There were significant indirect effects of fear of cancer recurrence on global health status through face [a1b1; 0.0394, Boot CIs 0.0025, 0.0819] and yield [a3b3; -0.1075, Boots CIs − 0.1657, -0.0557] but not for evasive [a2b2; 0.0235; Boots CIs − 0.057, 0.0508].

**Conclusions:**

Coping strategies are the most important mediating factors between fear of cancer recurrence and QOL among patients with prostate cancer. Our results support the proposed conceptual model, based on Lazarus’ and Folkman’s theory. Medical personnel need to develop corresponding intervention measures based on the different coping methods of patients, promote disease recovery, and improve postoperative quality of life.

## Introduction

Prostate cancer is a malignant tumour with a high recurrence rate, high mortality rate, and serious consequences for the health of elderly men. Its mortality rate is second only to lung cancer, and the majority of patients are elderly people. Surgical treatment is currently one of the most effective treatment methods for prostate cancer [1–2]. Postoperative patients with prostate cancer often experience various physical symptoms, such as urinary incontinence, leakage, bleeding, and pain, which greatly affect the patient’s physical and mental health [3]. Postoperative patients with prostate cancer need to address symptoms, treatment, dysfunction, comorbidities, and unpredictability regarding symptoms and disease progression, namely, fear of cancer recurrence. Fear of cancer recurrence is one of the psychological problems, defined as the fear that cancer may recur or progress in the same or other parts of the body. It is one of the most common psychological burdens experienced by cancer survivors, with a prevalence rate ranging from 39 to 97%, and it manifests as behavioural disorders, depressive syndrome, and social psychological distress [4–5].

In 1985, Lazarus and Folkman proposed the stress and coping model based on modern stress theory. They pointed out that coping is a continuous need for people to alleviate internal or external stress through conscious and behavioral efforts, as well as evaluating individual abilities. It is a process of relieving psychological pressure [6]. Wang’s research points out that there is a strong positive correlation between positive coping strategies and social psychological quality of life in patients with pulmonary hypertension [7]. Greer’s research shows that patients with little hope of recovery may adopt more surrender coping strategies [8]. There are also studies showing that the “avoidance” strategy seems to be beneficial for the mental and physical health of cancer patients [9], and “facing” is positively correlated with postoperative intestinal emptying time and analgesic dosage [10]. How patients with prostate cancer cope with these complications after surgery and what coping resources they have and use can impact their quality of life (QOL). Postoperative cancer patients note that physical limitations, symptoms, chronic conditions, and the progression of the disease all have a negative impact on their quality of life [11–12]. They try to cope with some of the negative issues to maintain their independence and a sense of normalcy in their lives [13]. The aim of this study is to understand the current situation of fear of recurrence, coping strategies, and quality of life in patients with prostate cancer after surgery and to analyse the path relationship between the three variables to provide a theoretical basis for improving the fear of disease progression in patients with prostate cancer after surgery.

**Fig. 1:**
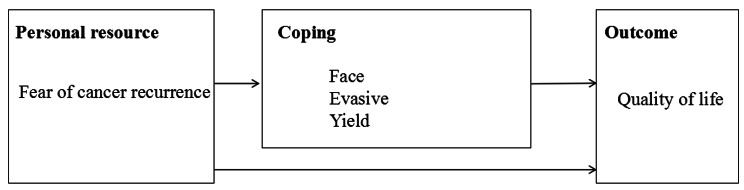
This chart is based on the medical coping mode. The arrows in the figure show the process of assuming the impact of the variables tested in this study. The Fear of cancer recurrence is assumed to influence the results directly and indirectly (through imitation)

We hypothesized that (a) fear of cancer recurrence negatively affects global QOL; (b) fear of cancer recurrence is related to coping strategies; (c) coping strategies are related to QOL; and (d) the relationships between fear of cancer recurrence and the global QOL are mediated by coping strategies (i.e., indirect effects of fear of cancer recurrence on QOL through coping) (Fig. 1).

## Materials and methods

### Design

The present multicentre cross-sectional study had a correlational design and included self-reported data from postoperative patients with prostate cancer.

### Study participants

The sample included patients from several comprehensive hospitals in urban areas. Eligible patients were (1) diagnosed with localized prostate adenocarcinoma and (2) treated with curative radical prostatectomy (RP) between 2021 and 2023. Patients were excluded if they received hormone therapy or adjusted their treatment plan. In January 2023, researchers identified eligible patients from the urology management follow-up patient database. After excluding the deceased patients, we sent the survey questionnaire to the surviving patients via WeChat in the form of an electronic questionnaire. Because 48 patients had incomplete questionnaire replies, 305 were included in the analysis. All participants provided written informed consent according to the Declaration of Helsinki. The study received research ethics committee approval by the Ethics Review Committee of the Army Military Medical University, on September 22, 2022 (202,243,001).

### Data collection

The data were collected from April 2023 to May 2023 using three self-reported questionnaires. In addition, data on personal characteristics such as age, gender, occupation, education level, marital status, fertility, course of illness, and economic situation were also collected.

#### Fear of cancer recurrence

We used the 12-item short version of the Fear of Progression Questionnaire (FoP-Q-SF) [13–14]. Among them, there are 8 items for family health and 4 items for social function, using the 5-point Likert scale, with a score range of 12–60 points. The higher the score is, the stronger the patient’s fear, and a score > 34 points indicates greater psychological dysfunction. Research has shown that this scale is highly correlated with the original FoP-Q scale (*r* = 0.92) and has good internal consistency (Cronbach’s alpha = 0.87).

#### Coping strategies

The Medical Coping Modes Questionnaire (MCMQ) was developed by Feifel in 1987 [15], and the Chinese version was translated and revised by Chinese scholar Shen Xiaohong into 20 items, including three dimensions: facing (8 items), avoiding (7 items), and yielding (5 items). Each item is scored using a 4-point Likert scale, with Items 1, 4, 9, 10, 12, 13, 18, and 19 being scored in reverse. The internal consistency of the three dimensions is 0.69, 0.60, and 0.76, respectively.

#### Quality of life

The QLQ-C30 (Questionnaire for Quality of Life Assessment in patients with cancer, version 3.0) is a tool for assessing quality of life in cancer patients, consisting of 30 questions subsequently transformed into 15 scales: five functional dimensions (physical, role, emotional, cognitive, and social), three symptom items (fatigue, nausea or vomiting, and pain), six single items (dyspnoea, sleep disturbance, appetite loss, constipation, diarrhoea, and financial impact) and a global health status/QOL scale [16]. Higher scores for global health status indicate better health. Conversely, higher scores for symptoms and functioning indicate worse health. Five functional scales and global health status items were selected as the main survey tools for this study.

### Data analysis

Descriptive statistics were used to describe the study variables, and Cronbach’s alpha coefficients were calculated for the questionnaires used. The counting data are described using frequency and composition ratio. Pearson correlation analysis was used to analyse the correlation between fear of illness, coping strategies, and quality of life.

To test our hypothesis about the direct and indirect effects of fear of cancer recurrence on psychological quality of life, we used a sequence multiple mediation model described by Hayes [17] and the process macro of SPSS. Point estimates unstandardized regression coefficients as well as bias-corrected and accelerated 95% confidence intervals (Boot CIs) are presented with the number of bootstrap samples set at 5000. Residuals were screened (histogram, normal P–P plot of regression residual, normal Q–Q plot of residual, and box-plots) and judged to be normally distributed. Data were checked for multicollinearity between independent variables using the variance inflation factor (VIF), and all VIF values were below 10 (ranging from 1.1 to 3.3), indicating no problem. Statistical analyses were performed using IBM SPSS Statistics 26. For all tests, the statistical significance level was set at *p* < 0.05.

## Results

### Demographics and clinical characteristics

The characteristics of the patients are presented in Table 1. There were 305 patients with ages ranging from 35 to 93 (mean age = 60.30, SD = 11.976). The average postoperative time was 10.26 months. Most of the patients were married or cohabitating. Forty-eight (22.4%) patients received only compulsory education, and 174 (81.3%) patients had sons. More than half of patients reported a heavy financial burden. The total FoP-Q-SF score of 305 postoperative prostate cancer patients was 34.3 ± 5.856, with approximately 41.6% of the patients scoring higher than 34 (a total score greater than 34 indicates psychological dysfunction) [18] (Table 2).

**Table 1:** Characteristics of the patients (*N* = 305)

Variable	*N* = 305 (%)
Age	
Mean (SD)	60.43 (11.879)
Range	35–93
Postoperative months(SD)	10.26(7.519)
Civil status	
Married/cohabitating	271 (88.9)
Single	34 (11.1)
Education level	
Compulsory school	120 (39.3)
Senior high school	134 (43.9)
University	51 (16.7)
Fertility circumstance	
Having a son	169 (55.4)
Without a son	136 (44.6)
Economic burden	
Very heavy	78 (25.6)
Heavy	68 (22.3)
Common	137 (44.9)
Lighter	22 (7.2)
Postoperative duration	
≤ 5	253 (83)
5–10	35 (11.5)
≥ 10	17 (5.6)

**Table 2:** Score of each variable (*N* = 305)

Variable	Number of items	Average score for each item	Total score
Fear of cancer recurrence	12	2.8 ± 0.488	34.3 ± 5.856
Coping strategy			
Face	8	2.3 ± 0.434	18.6 ± 3.851
Evasive	7	2.3 ± 0.381	16.3 ± 2.67
Yield	5	1.8 ± 0.519	9.0 ± 2.60
Quality of life	Number of items	Raw score	Standardized scores
Physical functioning	5	1.8 ± 0.520	68.4 ± 21.679
Role functioning	2	1.8 ± 0.705	71.9 ± 23.488
Emotional functioning	4	1.9 ± 0.637	66.3 ± 23.176
Cognitive functioning	2	1.9 ± 0.690	65.5 ± 27.598
Social functioning	2	1.9 ± 0.654	56.8 ± 25.881
Global Health Status Scale	2	4.4 ± 1.553	58.7 ± 28.881

### Bivariate correlations between main study variables

Face coping was positively correlated with emotional functioning, cognitive functioning, social functioning, global health status scale, and emotion-focused coping with problem-focused coping. Quality of life and face coping were negatively correlated with FoP-Q-SF (Table 3).

**Table 3:** Correlations between the main study variables and descriptive statistics (*N* = 305)

Variables	Fear	Coping strategy			Quality of life					
	1	2	3	4	5	6	7	8	9	10
Fear of cancer recurrence	1									
Face	0.32^*^	1								
Evasive	0.284^**^	0.209^**^	1							
Yield	0.251^**^	-0.224^**^	-0.066	1						
Physical functioning	-0.471^**^	-0.042	-0.435^**^	-0.251^**^	1					
Role functioning	-0.181^**^	-0.034	-0.044	-0.335^**^	0.401^**^	1				
Emotional functioning	-0.563^**^	0.164^**^	-0.204^**^	-0.340^**^	0.405^**^	0.183^**^	1			
Cognitive functioning	-0.392^**^	0.163^**^	-0.082	-0.244^**^	0.410^**^	0.126^*^	0,617^**^	1		
Social functioning	-0.412^**^	0.009	-0.127^*^	-0.235^**^	0.368^**^	0.271^**^	0.535^**^	0.422^**^	1	
Global Health Status Scale	-0.175^**^	0.269^**^	0.027	-0.445^**^	0.162^**^	0.236^**^	0.438^**^	0.106^**^	0.243^**^	1

*p < 0.05, **p < 0.01

Higher values indicate higher levels of the measured variables

### Mediator model

**Fig. 2:**
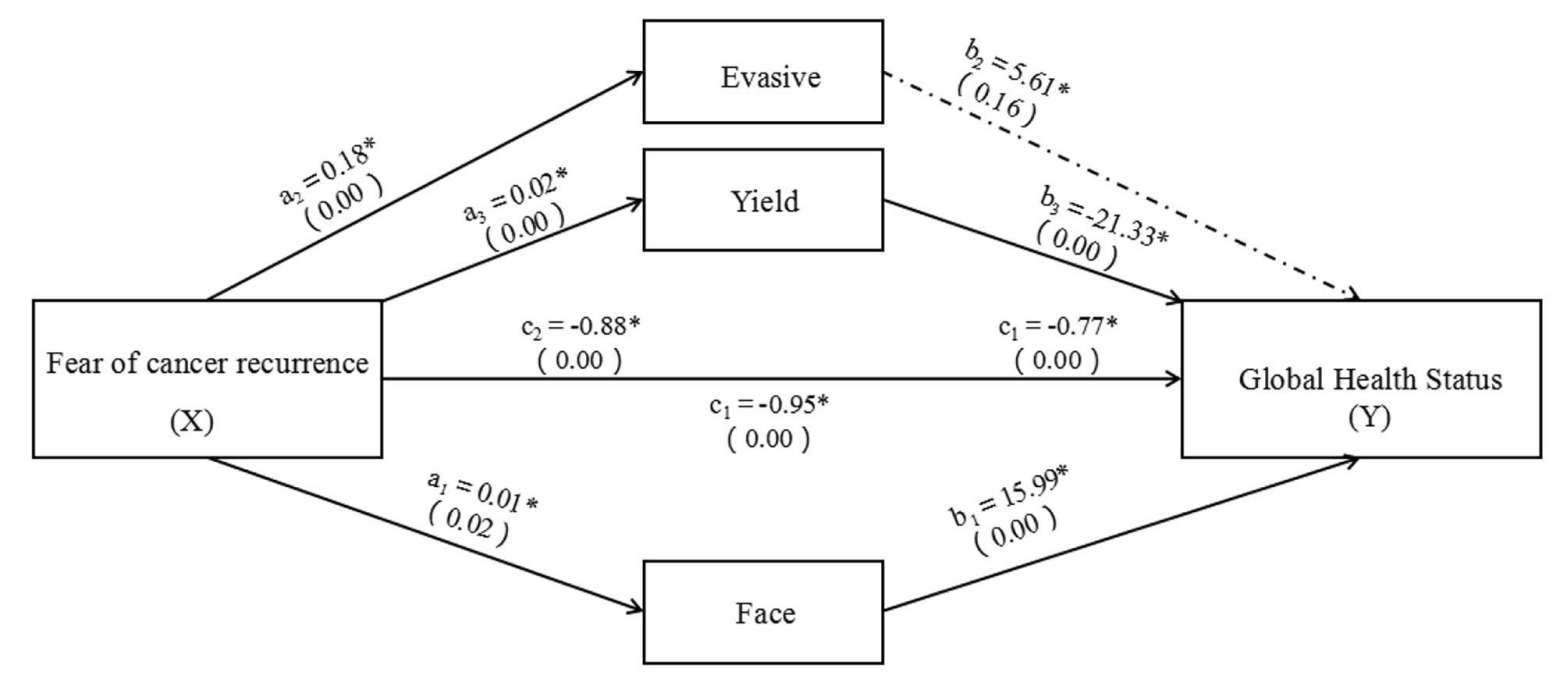
Structural equation model diagram of the mesomeric effect of strategy between fear of cancer recurrence and quality of life. The coefficient c is the total effect between X and Y while controlling for the three M. The variables are adjusted for age, gender, educational level, and economic status, *n* = 305. *p < 0.05 The serial multiple mediator model was tested using the PROCESS procedure for SPSS. Dotted lines represent nonsignificant paths

There were significant indirect effects of fear of cancer recurrence on global health status through face [a_1_b_1_; 0.0394, Boot CIs 0.0025, 0.0819] and yield [a_3_b_3_; -0.1075, Boots CIs − 0.1657, -0.0557] but not for evasive [a_2_b_2_; 0.0235; Boots CIs − 0.057, 0.0508], see Fig. 2. This indicates that face and yield partially mediate the relationship between fear of cancer recurrence and global health status.

## Discussion

Although the treatment methods for cancer continue to improve, there are still cases of recurrence or metastasis, which places a heavy burden on patients’ physical and mental health [19]. The total FoP-Q-SF score in postoperative patients with prostate cancer was 34.3 ± 5.856, indicating a high level of fear of disease progression. Patients exhibit significant psychological dysfunction, similar to other malignant tumour patients, such as lung cancer and breast cancer [20–21]. Of course, the research subjects included in this study were all postoperative patients with prostate cancer. Due to symptoms such as difficulty urinating and urinary incontinence, patients may experience negative psychological emotions such as inferiority, depression, and internal shame, which increase their psychological burden [22].

Our results support the proposed conceptual model, based on Lazarus’ and Folkman’s theory [6], suggesting that the coping strategies used and how effective they are perceived to mediate the relationship between fear of cancer recurrence and QOL among patients with prostate cancer. Evidence for the conceptual model adds knowledge to previous research, which has found relationships between fear of cancer recurrence and QOL in patients with prostate cancer but has not tested coping as a mediator [23].

This study demonstrates that fear of cancer recurrence can directly affect quality of life and can also affect it through different coping strategies. The direct impact of fear of cancer recurrence on quality of life is generated through different psychological states, and good family health and social function play an indispensable role [24]. Coping strategy is a means for individuals to handle stressful situations and maintain their balance during the face of stress and setbacks [9]. It can influence cancer patients to develop different psychological and behavioural states in response to stress events, and different coping strategies have varying effects on patients’ quality of life [25]. Research [12] has shown that positive coping strategies can better predict the quality of life of patients in terms of physiology, psychology, social relationships, and other aspects, while negative coping strategies can reduce their quality of life. Coping strategies play a mediating role in the fear of cancer recurrence and quality of life. The reason may be that the higher the patient’s fear of recurrence is, the higher the coping strategies, which can help them adopt a positive approach to face the disease and further improve their quality of life.

Our results emphasize the importance of mitigating the impact of yielding coping strategies, which enhance the negative impact of uncertainty on mental health [26]. Postoperative treatment and rehabilitation of prostate cancer is a lengthy process that requires not only the active efforts of medical staff but also the active participation of the patients [27]. Postoperative patients with prostate cancer not only have to bear the psychological impact of complications such as urinary incontinence and adverse reactions from treatment but they also worry about tumour recurrence and metastasis after surgery, which can easily lead to negative emotions such as anxiety, depression, and despair, ultimately leading to a decrease in their quality of life [28]. Negative coping styles can lower the patient’s self-management level. The submissive coping style is a negative one, which is not conducive to patients actively seeking medical assistance and understanding disease and self-management knowledge, resulting in insufficient knowledge and skills to manage their own healthy behaviour [29]. Therefore, the quality of life is reduced.

Finally, our research also shows that the path of avoidance as a coping style is not established, and the role of avoidance has been controversial. Faul’s research report [30] shows that avoidance is beneficial for cancer patients, as it can play a protective role in the patient’s body. However, more research [25] has characterized it as a negative coping style that is not conducive to the quality of life of patients. Thomsen [31] noted that avoidance has a positive effect on short-term stress, but if used for a long time, it is difficult to solve active problems.

### Limitations

This study has the following limitations. First, this study focuses on male prostate cancer patients, which limits the universality of our research results. Second, this is a multicentre cross-sectional study, so its causal relationship cannot be determined. Longitudinal research is needed to test the model and examine the relationships between variables of interest. Additionally, we use a retrospective approach to collect data, which may result in patient memory biases. Future research needs to validate our results in patients of different sexes and types of cancer. In addition, patients are almost all of Asian ethnicity, so it is necessary to test the model in populations with different sociodemographic and cultural backgrounds. Finally, future model testing should include patient assessment to explain how the relationship between uncertainty, coping strategies, and QOL is influenced by patient disease assessment.

## Conclusions

Coping strategies are the most important mediating factors between fear of cancer recurrence and QOL among patients with prostate cancer. This finding supports medical coping model theory. The results of the present study need to be further tested in other groups of prostate cancer and in longitudinal designs to generate knowledge on which to base more solid implications for clinical practice.

Patients’ fear of cancer recurrence not only directly affects their quality of life but also plays a role in quality of life through the mesomeric effect of different scoping strategies. Therefore, medical personnel are recommended to develop corresponding intervention measures based on the different coping pathways of patients, promote disease recovery, and improve quality of life.

## Data Availability

The datasets during and/or analysed during the current study are available from the corresponding author on reasonable request.
